# Supplementary material for: Analysis of PPARγ Signaling Activity in Psoriasis
Source: Int J Mol Sci. 2021 Aug 10;22(16):8603. doi: 10.3390/ijms22168603 (PMC8395241; doi:10.3390/ijms22168603)
Supplement: Supplementary file 1 [file ijms-22-08603-s001.zip › Supplemental materials_Analysis of PPARg signaling activity in psoriasis/Pathway models/Models images and html files/PPARG expressed in cells assotiated with psoriasis/225478.html]

T-cell


# CellType T-cell

|  |  |
| --- | --- |
| URN | urn:agi-ncimcelltype:C0039194 |
| Total Entities | 94 |
| Connectivity | 15408 |
| Name | T-cell |
| ObjectType | Cell |

---

|  |  |
| --- | --- |
| ChildConcepts | T-cell selection |
|  | T-cell function |
|  | T-cell interaction |
|  | T-cell adhesion |
|  | T-cell aggregation |
|  | T-cell proliferation |
|  | T-cell homeostasis |
|  | T-cell development |
|  | T-cell proliferative response |
|  | CD20+ T-cell |
|  | CD30+ T-cell |
|  | activated T-cell |
|  | anergic T-cell |
|  | senescent T-cell |
|  | gammadelta T-cell |
|  | exhausted T-cell |
|  | CD5- T-cell |
|  | CD5+ T-cell |
|  | Va24+ Vb11+ T-cell |
|  | alphabeta T-cell |
|  | effector T-cell |
|  | alloreactive T cell |
|  | mucosal-associated invariant T cell |
|  | intraepithelial lymphocyte |
|  | T-immunoblast |
|  | CD25+ T-cell |
|  | CD3+ T lymphocyte |
|  | rosette forming cell |
|  | T-cell infiltration |
|  | T-cell sequestration |
|  | T-cell localization |
|  | T-cell migration |
|  | T-cell fate |
|  | T-cell diapedesis |
|  | T-cell apoptosis |
|  | T-cell homing |
|  | T-cell clonal anergy |
|  | T-cell population |
|  | T-cell chemotaxis |
|  | T-cell transendothelial migration |
|  | T-cell survival |
|  | T-cell death |
|  | T-cell tethering |
|  | T-cell priming |
|  | T-cell mediated cytotoxicity |
|  | T-cell rolling |
|  | T-cell senescence |
|  | T-cell cytotoxicity |
|  | T-cell extravasation |
|  | T-cell growth |
|  | T-cell polarity |
|  | T-cell formation |
|  | T-cell motility |
|  | T-cell communication |
|  | T-cell distribution |
|  | T-cell transdifferentiation |
|  | T-cell motion |
|  | T-cell repertoire |
|  | T-cell fate commitment |
|  | T-cell phenotype |
|  | T-cell viability |
|  | T-cell contact |
|  | T-cell recognition |
|  | T-cell invasion |
|  | T-cell regeneration |
|  | T-cell spreading |
|  | T-cell fate determination |
|  | T-cell size |
|  | T-cell division |
|  | T-cell fate specification |
|  | T-cell ultrastructure |
|  | T-cell disruption |
|  | T-cell architecture |
|  | T-cell renewal |
|  | T-cell ratio |
|  | T-cell compartmentalization |
|  | T-cell destruction |
|  | T-cell replicative senescence |
|  | T-cell developmental process |
|  | T-cell lifespan |
|  | T-cell phagocytosis |
|  | T-cell damage |
|  | T-cell autophagy |
|  | T-cell morphogenesis |
|  | T-cell stress |
|  | T-cell elongation |
|  | T-cell agglutination |
|  | T-cell enlargement |
|  | T-cell volume |
|  | T-cell macrophage differentiation |
|  | T-cell swelling |
|  | T-cell transepithelial migration |
|  | T-cell structure |
|  | T-cell T-cell homeostasis |

---

|  |  |
| --- | --- |
| Pathway | Proteins/Ions Transporters -> Expression Targets in Lymphoid System and Blood |
|  | LDLR -> Expression Targets in Lymphoid System and Blood |
|  | TGF-beta Receptors Family -> Expression Targets in Lymphoid System and Blood |
|  | EGFR-> Expression Targets in Lymphoid System and Blood |
|  | Elevated Receptors -> Expression Targets in Lymph Node |
|  | Elevated Receptors -> Expression Targets in Lymphoid System and Blood |
|  | F2R/F2RL1 -> Expression Targets in Lymphoid System and Blood |
|  | CD Markers -> Expression Targets in Lymphoid System and Blood |
|  | VIPR1/VIPR2 -> Expression Targets in Lymphoid System and Blood |
|  | GPCRs Family -> Expression Targets in Lymphoid System and Blood |
|  | TNF Receptors -> Expression Targets in Lymphoid System and Blood |
|  | Immunoglobulin Superfamily -> Expression Targets in Lymphoid System and Blood |
|  | Receptor Tyrosine Kinase Family -> Expression Targets in Lymphoid System and Blood |
|  | Interleukin Receptors -> Expression Targets in Lymphoid System and Blood |
|  | CD40 -> Expression Targets in Thymus |
|  | C5AR1 -> Expression Targets in Lymphoid System and Blood |
|  | IgE Receptors -> Targets in Lymphoid System and Blood |
|  | Chemokine Receptor Family -> Expression Targets in Lymphoid System |
|  | NOTCH -> Expression Targets in Thymus |
|  | Elevated Receptors -> Expression Targets in Thymus |
|  | pathway 1 |
|  | PPARG expressed in cells assotiated with psoriasis |
|  | Neighbors of T-cell |
|  | Overview. Diseases of the blood (chapter 3) |
|  | 1\_HIV entrance to the host cell and viral reproduction |
|  | 1\_Antigen-presenting cells promote islet dysfunction and immune system activation in T1D |
|  | 2\_Defective tolerance of autoreactive T-cell in T1D |
|  | 1\_Decreased secretion of thyroid hormones |

---

|  |  |
| --- | --- |
| MedScan ID | 10000184 |

---

|  |  |
| --- | --- |
| Alias | immune T cells |
|  | T-cell |
|  | thymus-dependent lymphocyte |
|  | peripheral blood T cell |
|  | T-cells |
|  | T-lymphocytes |
|  | T-lymphocytic |
|  | peripheral blood T cells |
|  | peripheral blood T lymphocyte |
|  | thymus-dependent lymphocytes |
|  | peripheral blood T lymphocytes |
|  | T-lymphocyte |
|  | immune T cell |

---

|  |  |
| --- | --- |
| NCIm ID | C0039194 |

---
